# Milk yield, milk composition, and milk metabolomics of dairy goats intramammary-challenged with lipopolysaccharide under heat stress conditions

**DOI:** 10.1038/s41598-020-61900-8

**Published:** 2020-03-19

**Authors:** Ahmed A. K. Salama, Alexandra Contreras-Jodar, Samantha Love, Nabil Mehaba, Xavier Such, Gerardo Caja

**Affiliations:** 1 Group of Research in Ruminants (G2R), Department of Animal and Food Sciences, Universitat Autònoma de Barcelona, Bellaterra, Spain; 2 Chemokine Research Group, Institute of Infection, Immunity and Inflammation, University of Glasgow, Glasgow, UK; 3 Present Address: Department of Agri-Food Engineering and Biotechnology, Universitat Politècnica de Catalunya, Barcelona, Spain; 4 Present Address: Animal Welfare Program, Institut de Recerca i Tecnologia Agroalimentàries (IRTA), Girona, Spain

**Keywords:** Metabolomics, Molecular biology

## Abstract

Heat stress and mastitis are major economic issues in dairy production. The objective was to test whether goat’s mammary gland immune response to *E. coli* lipopolysaccharide (LPS) could be conditioned by heat stress (HS). Changes in milk composition and milk metabolomics were evaluated after the administration of LPS in mammary glands of dairy goats under thermal-neutral (TN; n = 4; 15 to 20 °C; 40 to 45% humidity) or HS (n = 4; 35 °C day, 28 °C night; 40% humidity) conditions. Milk metabolomics were evaluated using ^1^H nuclear magnetic resonance spectroscopy, and multivariate analyses were carried out. Heat stress reduced feed intake and milk yield by 28 and 21%, respectively. Mammary treatment with LPS resulted in febrile response that was detectable in TN goats, but was masked by elevated body temperature due to heat load in HS goats. Additionally, LPS increased milk protein and decreased milk lactose, with more marked changes in HS goats. The recruitment of somatic cells in milk after LPS treatment was delayed by HS. Milk metabolomics revealed that citrate increased by HS, whereas choline, phosphocholine, N-acetylcarbohydrates, lactate, and ß-hydroxybutyrate could be considered as putative markers of inflammation with different pattern according to the ambient temperature (i.e. TN vs. HS). In conclusion, changes in milk somatic cells and milk metabolomics indicated that heat stress affected the mammary immune response to simulated infection, which could make dairy animals more vulnerable to mastitis.

## Introduction

The negative effects of heat stress (HS) on the productivity of dairy animals in terms of milk yield, milk composition and milk quality are well documented^1,2^. Despite advances in cooling systems and environmental management, HS constitutes to be a significant cost for the dairy industry^3^. Goats, originated from hot and arid zones, are considered less sensitive to HS compared to cows. However, milk production losses have been reported in heat-stressed dairy goats, especially at early stages of lactation^4,5^.

The effect of HS on performance (e.g. milk yield, milk composition, feed intake, body temperature, respiratory rate) has been intensively evaluated in dairy animals^1,2^. However, only a few studies evaluated the omics of biofluids and tissues in animals exposed to HS such as cow’s blood plasma^6^, cow’s milk^7^, cow’s liver^8^ and goat’s urine^9^.

Besides the negative impact of HS on milk production, HS has been found to disrupt the immune function^5^. With regard to mammary immunity during HS, available data indicate that mammary immunity might be compromised by HS. Thompson *et al*.^10^ reported that cows without cooling during the dry period have higher incidence of mastitis in the ensuing lactation. At the systemic level, Contreras-Jodar *et al*.^5^ evaluated the transcriptomics of blood immune cells in heat-stressed goats and detected a decrease in the hematopoiesis and leukocyte diapedesis, which might compromise the innate and the adaptive immune response. They also reported a disruption in lipid metabolism of immune cells, which would significantly affect their functionality. Limited information is available on the metabolomics response of the mammary gland to infection under controlled HS conditions. The evaluation of milk metabolomics produced from goats under thermal-neutral (TN) or HS conditions with and without intramammary infection would lead to the detection of putative biomarkers for infection in both TN and HS conditions. As far as we know, milk metabolomics of TN and HS with and without mammary inflammation have not been evaluated in dairy animals. Obtained results would ultimately help in the development of new strategies to improve animal welfare and productivity under adverse conditions. Therefore, the aim of the present study was to evaluate the effect of both heat stress and simulated intramammary infection on milk yield, milk composition, and milk metabolomics in dairy goats.

## Materials and methods

### Animals, treatments and management conditions

Animal care conditions and management practices were approved by the Ethical Committee of Animal and Human Experimentation of the Universitat Autonoma de Barcelona (UAB), following procedures described in the Spanish and EU legislations (R.D. 53/2013, and Council Directive 2010/63/EU). Eight multiparous lactating Murciano-Granadina dairy goats (46 ± 2 kg of body weight; 101 ± 5 days in milk; 2.8 ± 0.1 L milk/d) from the herd of the Servei de Granges i Camps Experimentals of the UAB were used. Udders were confirmed to be healthy by culturing separate udder half milk onto Columbia 5% blood agar and assessing over 48 h.

Goats were divided into 2 balanced groups regarding milk yield and milk composition. The 2 groups were randomly assigned to 2 environmental treatments for 15 d. Treatments were: 1) thermal-neutral (TN; 15 to 20 °C and 45% relative humidity), and 2) heat stress [HS; 12-h day (from 08:00 to 20:00) at 35 °C and 40% relative humidity, and 12-h night (from 20:00 to 08:00) at 28 °C and 40% relative humidity]. In each environment treatment, one udder half was injected with *E. coli* lipopolysaccharide (LPS) or with 0.9% saline (CON) at 08:00 on day 12 after milking. Consequently, there were 4 treatment combinations: TN-CON, TN-LPS, HS-CON, and HS-LPS. The LPS udder halves aseptically received 10 μg *E. coli* LPS (O55:B5; Sigma-Aldrich, St Louis, MO) dissolved in 2 mL sterile 0.9% saline (0.9% NaCl; Braun; Barcelona, Spain), whereas the CON halves were administered with 2 mL saline. Before injection teat openings were disinfected with 70% alcohol.

For the TN goats, the temperature was maintained at 15 to 20 °C with the help of electric heater equipped with a thermostat (3.5 kW; General Electric, Barcelona, Spain) when necessary. The HS goats were kept in a 4 × 6 × 2.3 m climatic chamber (Euroshield, ETS Lindgren-Euroshield Oy, Eura, Finland) provided with a temperature and humidity controlling system (CAREL Controls Ibérica, S.L., Barcelona, Spain). A continuous 90 m^3^/h air turnover was maintained throughout the experiment. Temperature-humidity index (THI) was calculated according to NRC^11^ and was THI_TN_ = 59 to 65 and THI_HS_ = 83-day, 74-night.

Goats had a 2-wk pre-experimental period under TN conditions for the adaptation to the diet and experimental conditions. The averages of daily feed intake (2.65 ± 0.11 kg) and milk yield (2.83 ± 0.20 kg) during the last 3 days of the adaptation period were similar (*P* = 0.653) for goats assigned to TN and HS treatments. Photoperiod was maintained constant at 12-12 h light-dark (08:00 to 20:00) and data of environmental temperature and humidity were recorded every 10 min using 2 data loggers (Opus 10, Lufft, Fellbach, Germany).

The total mixed ration was distributed individually to each goat once daily after the a.m. milking and adjusted at 30% orts based on the previous day intake. The ration was formulated to cover requirements using INRAtion 4.07 software and consisted of (as fed) alfalfa hay 60.4%, ground barley grain 15%, beet pulp 9.1%, ground corn grain 7.5%, soybean meal 3%, sunflower meal 3%, molasses 1%, salt 0.6%, sodium bicarbonate 0.2%, and vitamin-mineral corrector for goats 0.2%. The ration contained (on DM basis) 15.1% CP, 39.3% NDF, 28.6% ADF, and 1.54 Mcal NE_L_. Mineral and vitamin blocks as well as water were freely available for each goat.

Goats were milked twice daily (08:00 and 16:00 h) using a portable milking machine (Westfalia Seperator Iberica SA, Granollers, Spain) set at 42 kPa, 90 pulses/min, 66% pulsation ratio. No udder preparation was done before milking. After milking teats were dipped in iodine solution (P3-io shield; Ecolab Hispano-Portuguesa S.L., Barcelona, Spain).

### Measurements, sampling, and analyses

Rectal temperatures (RT) and respiratory rates (RR) were recorded daily at 0800, 1200, and 1700 h. The RT was measured by a digital clinical thermometer (ICO Technology, Barcelona, Spain; range, 32 to 43.9 °C; accuracy, ±0.1 °C). The RR was calculated as the number of flank movements during 60 s. From d 12 to 15, RT and RR were measured at 0, 4, 8, 12, 24, 48 and 72 h after the intramammary LPS challenge.

Milk yield (kg/d) was daily recorded at each milking. Feed intake was calculated daily by the difference between the weight of the feed offered and the weight from the refusals using an electronic scale (model Fv-60K; A&D Mercury PTY, Thebarthon, Australia; accuracy, ±20 g). Water consumption was daily measured by an electronic scale (model JC30; JC Compact, Cobos Precision, Barcelona, Spain; accuracy, ±10 g). Trays with saw dust were put below the drinking troughs and weighed to take into account water wastes. Feed samples were collected daily, composited, and analysed (AOAC^12^).

For the LPS challenge test, milk yield for each udder half was weighed at the regular milking schedule from d 12 to 15. Milk samples from each udder half were collected at 0, 2, 4, 6, 8, 10, 12, 24, 36, 48 and 72 h after LPS challenge for composition. The volumes of sampled milk collected were included in total milk yield at each time point. Milk samples were collected and preserved with an antimicrobial tablet (Bronopol, Broad Spectrum Microtabs II, D&F Control Systems, San Ramon, CA) at 4 °C until analysis. Milk contents of total solids, fat, protein, lactose, and SCC were determined using Milkoscan (MilkoScan FT2 - infrared milk analyzer, Foss 260, DK-3400 Hillerød, Denmark) and an automatic cell counter (Fossomatic 5000, Foss Electric, Hillerød, Denmark) previously calibrated for goat milk. For milk metabolomics, additional milk samples (10 mL) without preservatives were collected at 0, 4, 6, 12, and 24 h after the LPS challenge and frozen at −80 °C until metabolomics analysis.

### Sample preparation and nuclear magnetic resonance spectroscopy procedures

Milk samples (n = 80) were prepared for metabolomics analysis according to Beckonert *et al*.^13^. Briefly, a phosphate buffer (pH 7.4) solution was prepared with Na_2_HPO_4_, NaH_2_PO_4_, NaN_3_, and D_2_O (Sigma-Aldrich Merck; Darmstadt, Germany). The solution was thoroughly shaken and left in a Clifton sonicator (Nickel Electro, Weston-super-Mare, United Kingdom) at 40 °C until the salts were dissolved.

After thawing at room temperature, 4 mL of milk were transferred to a filtration tube (Amicon Ultra-4, membrane PLGC Ultracel-PL, 10 kDa) and centrifuged at 22 °C for 20 min at 5,000 × *g* in a swing-bucket rotor (Hettich, Tuttlingen, Germany). Then, 400 μL of the ultra-filtrated milk were transferred into Eppendorf tubes and mixed thoroughly with 200 μL of cold phosphate buffer solution. Then, 550 μL of the final mixture were transferred into 5 mm-NMR tubes (VWR International, Eurolab, Barcelona, Spain). The prepared NMR tubes were immediately placed in ice and sent for NMR analysis.

^1^H NMR Spectra were acquired on a Bruker Avance-III spectrometer (Bruker BioSpin, Rheinstetten, Germany) operating at a proton NMR frequency of 600 MHz ^1^H and a temperature of 298°K controlled by BCU-extreme regulator. A 5 mm Triple Resonance Broadband Inverse (TBI) probe with *z*-gradients and inverse detection was controlled by TopSpin 3.1 software (Bruker, Germany). One-dimensional ^1^H NMR spectra were obtained using a one-dimensional nuclear overhauser enhancement spectroscopy pulse sequence. All data were collected with a spectral width (*δ*) of 12.0 ppm, and 0.3 Hz of exponential line broadening was applied for the Fourier Transform of the raw data. All NMR spectra were phased and baseline corrected using TopSpin 3.1 software.

### Statistical analyses

#### Thermophysiological and lactational performance data

The PROC MIXED for repeated measurements of SAS (version 9.1.3; SAS Institute Inc., Cary, NC) was used. For performance data (day 1 to 11), the statistical model contained the fixed effects of environmental treatment (TN and HS), day, measuring hour (for RT and RR), the random effect of the animal and interactions of environmental treatment × day; environmental treatment × measuring hour; day × measuring hour, environmental treatment × day × measuring hour, and the residual error. The interactions of day × measuring hour and environmental treatment × day × measuring hour were not significant (*P* > 0.20) and were excluded from the model.

For the LPS challenge period (d 12 to 15), the statistical model contained the fixed effects of environmental treatment (TN and HS), mammary challenge (CON and LPS), time after challenge (0, 2, 4, 6, 8, 10, 12, 24, 36, 48 and 72 h), interactions of environmental treatment × mammary challenge, environmental treatment × time after challenge, mammary challenge × time after challenge, and environmental treatment × mammary challenge × time after challenge; the random effect of udder half nested within the animal; and the residual error. Logarithmic transformations (log_10_) of SCC values were used in the statistical analysis. Differences between least squares means were determined with the PDIFF option of SAS.

### NMR data processing and analysis

Imprecisions in chemical shifts could occur due to differences in temperature, pH, ionic strength among others^14^. Therefore, each dataset was uniformly divided into bins of 100 by increasing the interval width from 0.0003 to 0.0300 ppm using the R software^15^ (version 3.2.3). Then, the raw ^1^H NMR spectral data were edited by excluding the regions outside the chemical shift (*δ*) range of 8.0 to 0.5 ppm, and also the residual peak of the imperfect water suppression (*δ* = 5.0 to 4.6 ppm).

A principal component analysis was performed without considering the class information to check for possible outliers. Then, Partial Least Squares-Discriminant Analysis (PLS-DA) multivariate analysis with leave-one-out cross validation was performed on the datasets using pls package of R software^16^. The optimum number of latent variables for each PLS-DA model was chosen by plotting the root mean square error of prediction of the cross validation against the number of latent variables. The minimum indicated the number of factors that minimize the error of prediction and the number of latent variables where chosen following the minimum error prediction under the maximum parsimony to build the model. Model strength was assessed using both R^2^ and Q^2^ statistical parameters. While R^2^ values report the total amount of variance explained by the model, the Q^2^ reports model accuracy as a result of cross-validation. Aside from its theoretical maximum of 1, an empirically inferred value of Q^2^ ≥ 0.4 is considered acceptable for a biological model^17^. The resulting Q^2^ statistic was compared to a null distribution to test model significance (*P* < 0.05).

Interpretation of multivariate analysis was performed through scores and loadings plots according to its contribution to the separation between groups. For the identification of possible biomarkers, PLS-DA loadings were sorted by its absolute value, with the greater ones being the metabolites responsible of the separation between experimental groups.

Chemical shifts linked with the highest loading values found in PLS-DA were annotated for metabolite assignment. Furthermore, the important metabolites were also statistically analysed using a mixed model for repeated measurements containing the fixed effects of environmental treatment (TN vs. HS), mammary challenge (CON vs. LPS), sampling hour (0, 4, 6, 12 and 24 h) and their interactions, and the random effect of udder half nested within the animal using nlme package of R software. The *P* values were corrected for multiple comparisons using the multcomp package of R. Metabolomic profile at 0 h did not vary between treatment groups (Q^2^ = −0.50), and therefore a covariate was not necessary. The chemical shifts were assigned to their corresponding metabolites according to Sundekilde *et al*.^18^ and the Human Metabolome Database^19^.

## Results and discussion

### Effects of heat stress on physiological and productive performance

An increase (*P* < 0.001) in RT and RR was observed in HS goats throughout the day in accordance with the increment in THI. However, measuring hour had no effect on RT or RR in TN goats (Table 1). Maximum RT difference (+1.65 °C; *P* < 0.001) and RR (+141 breaths/min; *P* < 0.001) between HS and TN goats occurred at 1700 h (Table 1). Increased respiration rate under HS conditions is a known mechanism for dissipating the thermal load by evaporation.

**Table 1:** Rectal temperature and respiratory rate of dairy goats exposed to thermal neutral (TN; n = 4) and heat stress (HS; n = 4) conditions from day 1 to 11 (before the LPS treatment).

Item	Treatment		SEM	Effect (*P*- Value)		
	TN	HS		Treatment	Hour	T × H
Rectal temperature, °C						
0800 h	38.54	39.25^c^	0.18	0.001		
1200 h	38.57	40.02^b^	0.18	0.016		
1700 h	38.80	40.46^a^	0.18	0.016		
Average	38.64	39.91	0.12	0.001	0.001	0.001
Respiratory rate, breaths/min						
0800 h	34	81^c^	10	0.001		
1200 h	39	171^b^	10	0.001		
1700 h	46	187^a^	10	0.001		
Average	40	147	7	0.001	0.001	0.001

SEM = standard error of the mean. T × H = treatment × measuring hour interaction. ^a–c^ = means with different superscripts within the same column differ (*P* < 0.05).

The DM intake decreased by 27% in HS (*P* < 0.05) when compared to TN goats (Table 2). In contrast, water consumption increased by 68% (*P* < 0.001) under HS conditions. Obtained results agree with those reported for the same breed of dairy goats under similar HS conditions^4,5^. The decrease in feed intake is a highly-conserved response among species when environmental temperature increases and occurs in an attempt to reduce animal’s metabolic heat production^20^. Increased water intake under HS conditions is mainly used for boosting latent heat loss by evaporation (e.g., sweating and panting)^21^.

**Table 2:** Feed intake, water consumption and milk yield of dairy goats exposed to thermal neutral (TN; n = 4) and heat stress (HS; n = 4) conditions from day 1 to 11 (before the LPS treatment).

Item	Treatment		SEM	Effect (*P*- Value)		
	TN	HS		Treatment	*day*	*Treatment* × *day*
Feed intake, kg DM/d	2.59	1.87	0.24	0.025	0.197	0.440
Water consumption, kg/d	6.0	10.1	1.1	0.001	0.281	0.404
Milk yield, kg/d	2.75	2.18	0.34	0.099	0.068	0.988

SEM = standard error of the mean.

Heat-stressed goats tended to decrease (*P* < 0. 10) milk yield by 21% compared to the TN (Table 2). Among livestock species, and despite the marked differences between breeds within the same species, goats were generally reported to have the most tolerance to elevated ambient temperatures^22^. Milk yield losses in goats (−3 to −13% at THI = 80 to 85)^1^ are less pronounced compared to sheep (−20% at THI = 72 to 75)^23^ and cows (−27 to −33% at THI = 73 to 82)^24^.

These thermophysiological and lactational performances clearly indicates that at d 11, goats were under significant HS, at which time the intramammary LPS challenge was carried out.

### Responses to intramammary LPS challenge under thermo-neutral and heat stress conditions

The intramammary infusion of LPS in one half-udder while keeping the other half as control has been used by us and others to evaluate mammary immunity in sheep^33^ and cows^25–29^. It mimics responses to natural mastitis but without causing a true infection^26^. The LPS model used in the current study successfully simulates controlled inflammation over all the experimental animals (same LPS and same dose), but in contrary to what happens in natural mastitis, there are no live pathogens able to multiply and adhere to the tissue. Additionally, metabolites secreted directly by microorganisms into the milk during natural mastitis are missing in the LPS model, and therefore metabolites evaluated in the current study are solely secreted from immune and mammary cells.

One of the main questions addressed by the current study was whether heat stress alters responsiveness of lactating dairy goats to intramammary endotoxin challenge. To answer this question, both TN and HS goats received LPS in one udder half, whereas the other udder half was injected with saline on d 12.

**Figure 1:**
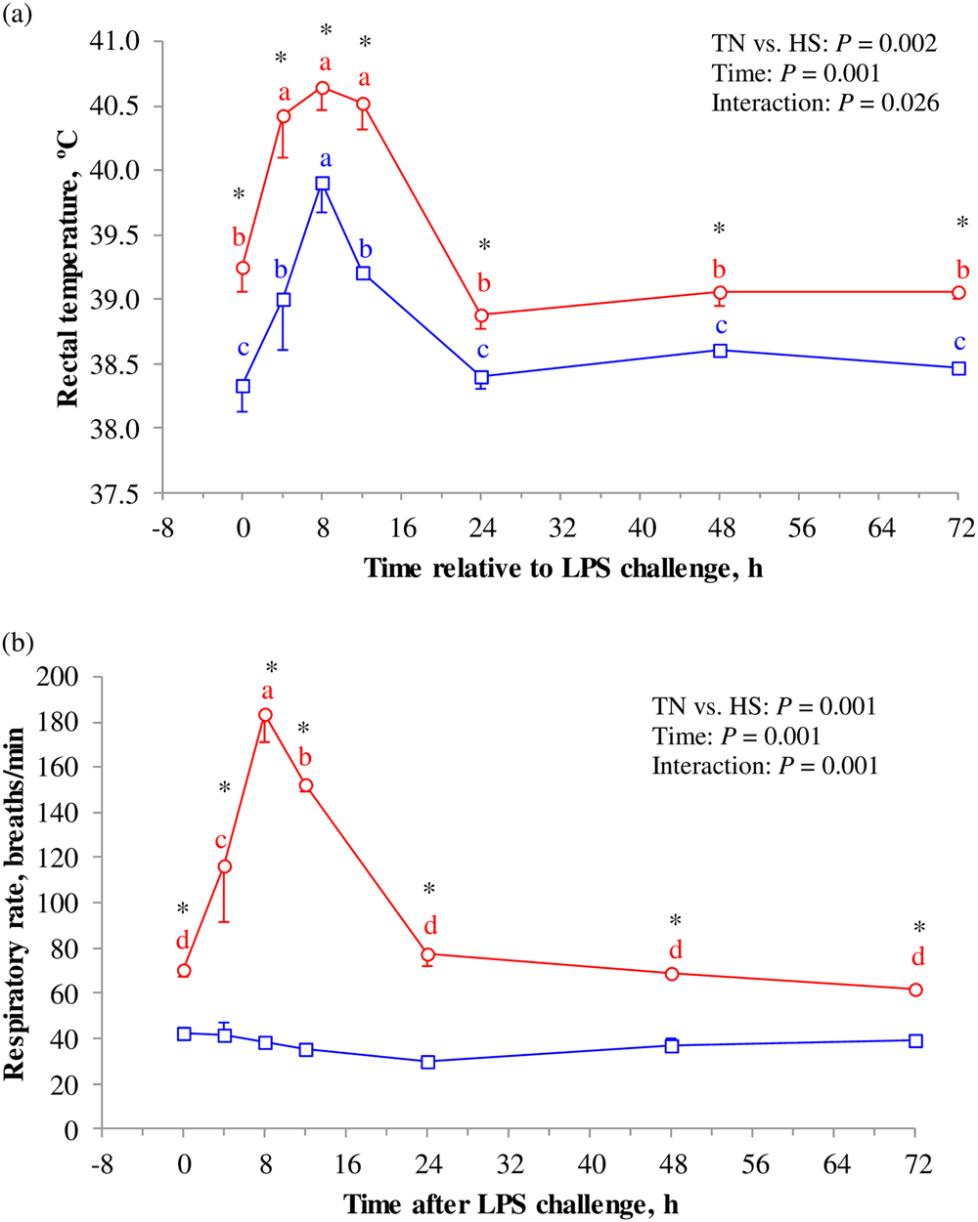
Hourly means and SEM for rectal temperature (**a**) and respiratory rate (**b**) following the intramammary injection of 10 μg *E. coli* endotoxin in thermo-neutral (TN; n = 4; ) and heat stress (HS; n = 4; ) goats. At 0 h (08:00 h), ambient temperatures were 15 and 28 °C for TN and HS goats, respectively, which explains the greater rectal temperature and respiratory rate in HS goats at 0 h before the administration of LPS. ^*^Indicates differences (*P* < 0.05) between TN and HS at each time point. ^a-d^Time points within each treatment (TN or HS) with different superscript differ (*P* < 0.05).

There was an interaction (*P* < 0.05) between the heat treatment and time relative to LPS challenge for rectal temperature and respiratory rate (Fig. 1). Rectal temperatures in HS goats were greater (*P* < 0.05) than in TN at all-time points. The local treatment with LPS in one udder-half resulted in systemic increment (*P* < 0.01) in rectal temperature (Fig. 1). In both TN and HS goats, rectal temperatures at 4, 8 and 12 h after challenge were greater (*P* < 0.01) than at 0 h. From 24 h onwards, rectal temperatures returned to 0 h values. Similarly, intramammary injection of endotoxin in one udder quarter results in significant increment in rectal temperatures at 5 to 8 h post challenge (40.5 to 41.1 °C) in dairy cows^27–29^. Body temperature has immunomodulatory functions^30^ as hyperthermia increases cytokine and nitric oxide production after the challenge by endotoxins^31^.

Rectal temperature in TN goats at 8 h (39.9 °C) was greater (*P* < 0.05) than values at 4 (39.0 °C) or 12 h (39.2 °C), with no differences between 4 and 12 h values. The RT recorded at 4, 8 and 12 h post challenge in TN goats were greater than the normal daily values recorded in the days before the LPS challenge (38.5 to 38.8 °C; Table 1). In HS animals, RT at 4 (40.4 °C), 8 (40.7 °C), and 12 h (40.5 °C) post challenge were not different from each other, but were greater (*P* < 0.001) than at other time points, including 0 h. Additionally, these RT values in HS goats at 4, 8 and 12 h after LPS injection (corresponding to 12:00, 16:00, 20:00) were similar to the normal RT values recorded during the days before the LPS challenge (40.0 and 40.5 °C at 12:00 and 17:00, respectively; Table 1). The high ambient temperatures normally recorded in HS goats at 12:00 to 17:00 precluded the detection of the increment in RT at 4 to 12 h after LPS injection. Consequently, the ambient temperature should be considered for the interpretation of data when dealing with detection of infections. Thus, under TN conditions an increase in body temperature would indicate a febrile response to an infection, whereas under HS conditions the febrile response could be masked by a greater heat load.

Respiratory rate at all time points was greater (*P* < 0.05) in HS than in TN goats (Fig. 1). From 0 to 8 h rectal temperature increased by similar magnitude in TN (+1.57 °C) and HS (+1.40 °C) goats, However, respiratory rate did not change in TN, but increased (*P* < 0.001) by 115 breaths/min in HS goats. Similar to our results in TN goats, intramammary infusion with endotoxin resulted in no change in respiratory rate or even a slight decrease^27^. However, other researchers reported increases in respiratory rate after intramammary challenge by LPS^29^. In our HS goats, the increment in respiratory rate at 4 to 12 h after LPS administration (12:00 to 20:00) is a consequence of the increment in the ambient temperature (ambient temperature was 28 °C from 20:00 to 08:00, and 35 °C from 08:00 to 20:00) rather than the febrile response to LPS.

With regard to milk yield, no significant interaction between LPS and temperature effects (*P* = 0.697) was detected. Furthermore, milk yield did not vary (*P* = 0.219) between LPS (1077 mL/d) and CON udder-halves (1240 mL/d). However, an interaction between time after challenge and LPS treatment was detected (*P* < 0.05) due to the fact that milk yield in CON half-udders was greater (*P* < 0.05) at 24 h (1248 mL/d) than at 0 h (1080 mL/d) and steadied thereafter, while milk yield in LPS halves did not vary throughout the challenge period (1073 mL/d on average).

**Figure 2:**
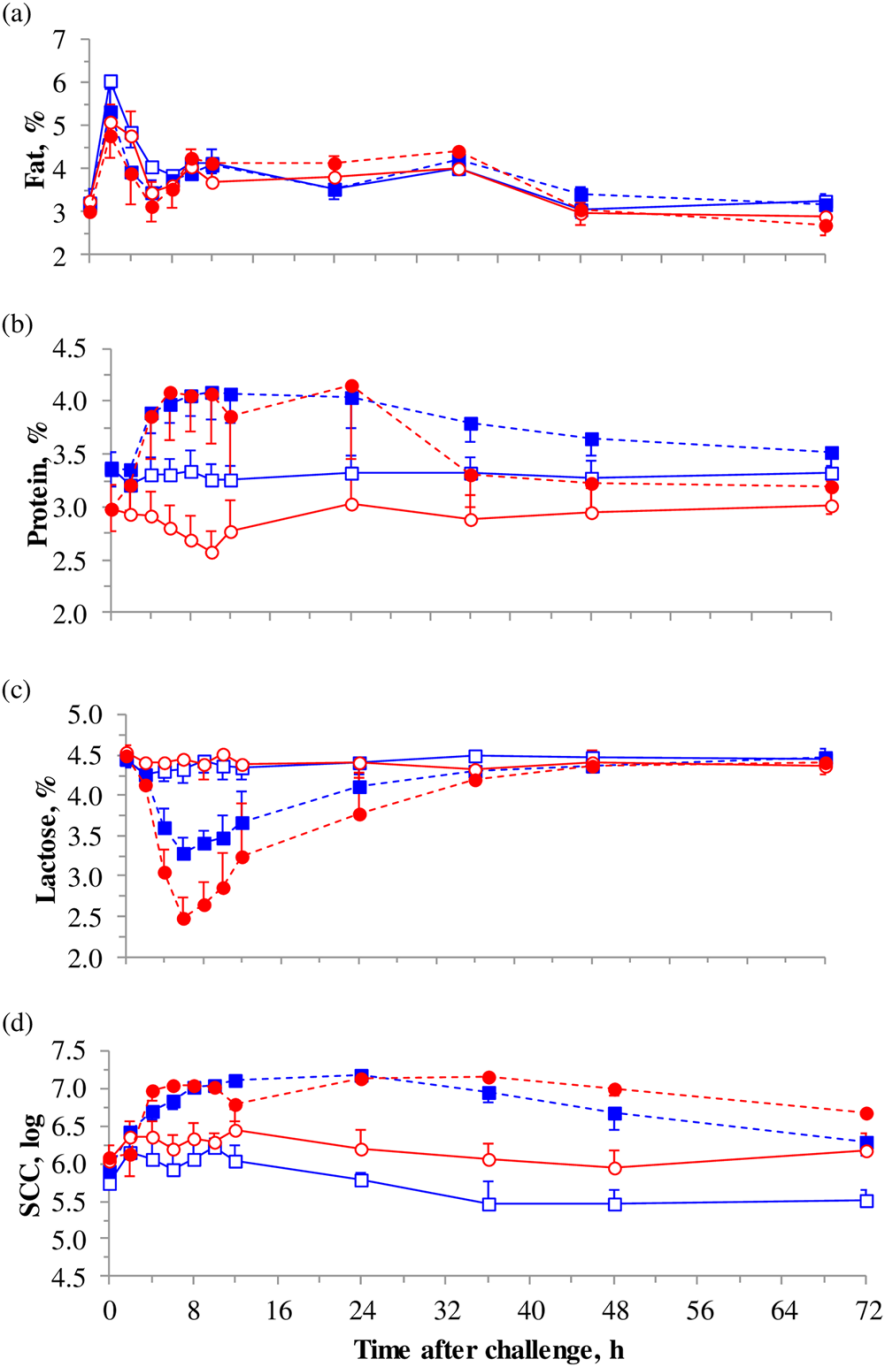
Hourly means and SEM for milk fat (**a**), protein (**b**), lactose (C), and SCC (**d**) following intramammary injection of endotoxin in dairy goats maintained under thermal neutral (TN; n = 4) or heat stress (HS; n = 4) conditions for 15 days. On day 12, one half-udder in each TN and HS goat was administered with 10 μg *E. coli* lipopolysaccharide (LPS), whereas the other half was injected with saline as the control (CON). This resulted in 4 treatment combinations: TN-CON (), TN-LPS (), HS-CON (), and HS-LPS ().

Milk fat was not affected by ambient temperature (*P* = 0.394) or endotoxin infusion (*P* = 0.505) (Fig. 2a). The initial peak of milk fat at 2-h sampling (*P* < 0.01) is most likely due to the fact that milk collected a very short time after milking is mainly alveolar milk which is rich in fat content^32^. At 6-h sampling onwards, the proportion of cisternal milk (which is poorer in fat content) is greater, which explains the reduced fat concentration.

There were significant temperature × time (*P* < 0.01), LPS × time (*P* < 0.001), and temperature × LPS × time (*P* < 0.05) interactions for milk protein (Fig. 2b). The LPS challenge resulted in greater milk protein (3.72 vs. 3.08% for LPS and CON udder-halves, respectively; *P* < 0.01), whereas HS did not affect the milk protein (*P* = 0.188). Greater milk protein contents in LPS challenged mammary glands were also observed in dairy ewes^33^ and cows^34^, which has been attributed to the influx of blood-borne proteins^34^. From 2 to 24 h the increment in milk protein caused by LPS injection was more marked in HS (+1.08 points) than in TN goats (+0.63 points). Additionally, the difference between CON and LPS halves in milk protein started to be significant (*P* < 0.01) at h 2 in HS goats, whereas in TN goats the difference started to be significant (*P* < 0.05) later at h 4. This finding could indicate different timing in the inflammation process under HS conditions. Significant difference in milk protein between CON and LPS halves in both TN and HS disappeared from h 36 onwards.

There were significant temperature × time (*P* < 0.001), LPS × time (*P* < 0.001) and temperature × LPS × time (*P* < 0.01) interactions for milk lactose (Fig. 2c). Lactose content was not affected by HS (*P* > 0.05), but LPS challenge induced a dramatic decrease (*P* < 0.001) in lactose concentration with the lowest levels observed at 6 h post-challenge (−1.1 points in TN-LPS and −2.0 points in HS-LPS). Lactose levels in TN-LPS and HS-LPS were recovered at 24 and 36 h post challenge, respectively when compared to their correspondent CON halves. This could indicate that mammary tight junctions in HS goats could have remained opened for longer time than in TN animals. Compared to TN-LPS, HS-LPS had or tended to have lower milk lactose at 4 to 8 h after LPS infusion (*P* = 0.011 to 0.072). The decrease in lactose content in LPS challenged mammary glands was also observed in dairy ewes^33^. The drop in lactose in milk is an indicator of tight-junction leakiness^35^ since lactose partially moves to blood. LPS is the specific ligand of TLR4 (toll-like receptor-4) stimulating the translocation of NFkB transcription factor from the cytoplasm to the nucleus^36^. The activation of NFkB pathway increases tight junction permeability through expression changes of claudins in the mammary gland^37^. The higher losses of milk lactose detected in HS-LPS can be explained by greater tight junction leakiness, since HS has been shown to compromise tight junction integrity in other tissues such as the gastrointestinal barrier^9,38^.

Heat stress had no effect on SCC (*P* = 0.132), but LPS challenge induced acute inflammation that was reflected by an increase in log_10_ SCC from 6.04 to 6.78 (*P* < 0.001) on average (Fig. 2d). The SCC is accepted worldwide as an indicator of immune response of the mammary gland to invading microorganisms. The increment in SCC following intramammary LPS challenge is caused by the infiltration of blood immune cells into the mammary gland^28,29^. By 72 h, SCC values in LPS halves were still greater than their own initial values at 0 h in TN (*P* < 0.08) and HS (*P* < 0.001) goats. The half-life of LPS in the mammary gland is not known. However, previous studies in dairy cows indicated that immune-stimulating effects of intramammary LPS last longer than 12 h even if the cows are milked 12 h after LPS administration^39^. In the current study, the effect of LPS on SCC lasted for at least 72 h even though the goats were milked 6 times after the administration of LPS.

Resident somatic cells have a long storage period (milking interval) in the udder and during this time cells ingest fat globules and casein^40^, resulting in reduced phagocytic and bactericidal activities, and consequently, the mammary gland immunity is impaired^41^. Therefore, the immediate entrance of new blood cells to the mammary gland is primordial to face infections. The recruitment of new somatic cells (indicated by time at which SCC increased with regard to 0 h) was faster in TN than in HS halves. Compared to 0 h values in LPS halves, log_10_ SCC increased at 2 h by 0.54 units in TN halves (*P* < 0.001) and remained higher than the 0 h values until 48 h (*P* < 0.01) and 72 h (*P* < 0.10). On the other hand, log_10_ SCC in HS-LPS did not increase from 0 to 2 h (only +0.05; *P* = 0.758), but increased at 4 h onwards (*P* < 0.01). This result can indicate a faster immune response in TN goats with earlier entrance of new cells with more phagocytic capacity.

### Effect of heat stress on milk metabolome

**Figure 3:**
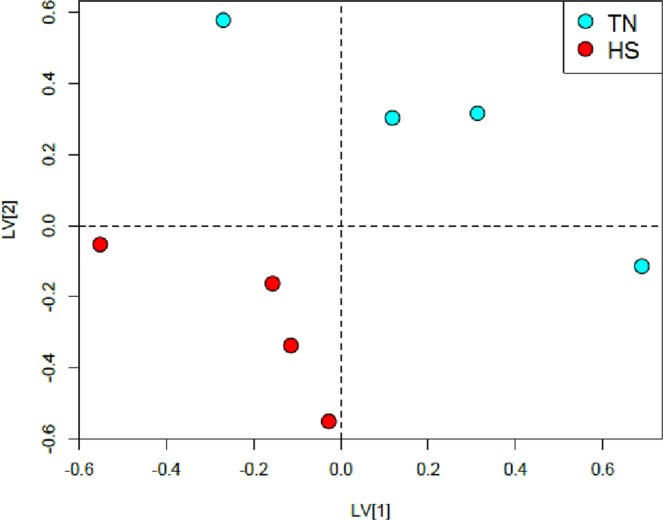
Partial Least Squares-Discriminant Analysis (PLS-DA) scores plot of ^1^H NMR metabolomics spectra of milk produced from udder halves of goats maintained under thermo-neutral (TN; n = 4) or heat stress (HS; n = 4) conditions for 15 days. Milk samples were collected before the administration of LPS on day 12.

The metabolomic profile was analysed using milk of HS and TN goats from the CON udder halves at 0 h. A principal component analysis was initially applied to all data, and no outliers were detected based on the principals of Hotelling’s T^2^ (95% interval of confidence). Consequently, PLS-DA was applied to identify the key metabolites responsible of the differences in milk metabolome between TN and HS dairy goats. The PLS-DA scores plot showed a clear separation between HS and TN datasets (Fig. 3). The cross-validation (first 2 components) gave *R*^2^_*x*_ = 0.36, *R*^2^_*y*_ = 0.60 and *Q*^2^ = 0.24. The *R*^2^ and *Q*^2^ values in the model were significantly higher than in the random model (*P* < 0.01), although *Q*^2^ did not reach the acceptable value for a recommended biological model (i.e. ≥0.40^17^) probably because of the small sample size used in the present comparison (4 goats TN vs. 4 goats HS). However, the principal objective of the current study was to determine whether the response of the mammary gland to inflammation varies according to ambient temperature (i.e. TN vs. HS). This objective was achieved by the comparison of both udder halves (one control and one treated with LPS) within the same animal (see the following section), which eliminates the variation due to animal, and makes it easier to detect differences with fewer animals.

The top-ranking chemical shifts responsible for discriminating milk metabolome between TN and HS goats were *δ* 2.55, 2.52 and 2.65 ppm. These chemical shifts corresponded to citrate, which was greater in milk of HS goats (log_2_ FC = 2.89; *P* < 0.01). A recent report agrees with our results, where citrate has been also described as a biomarker for HS in cow’s milk^7^.

### Effect of intramammary LPS challenge on milk metabolome under thermal-neutral and heat stress conditions

**Figure 4:**
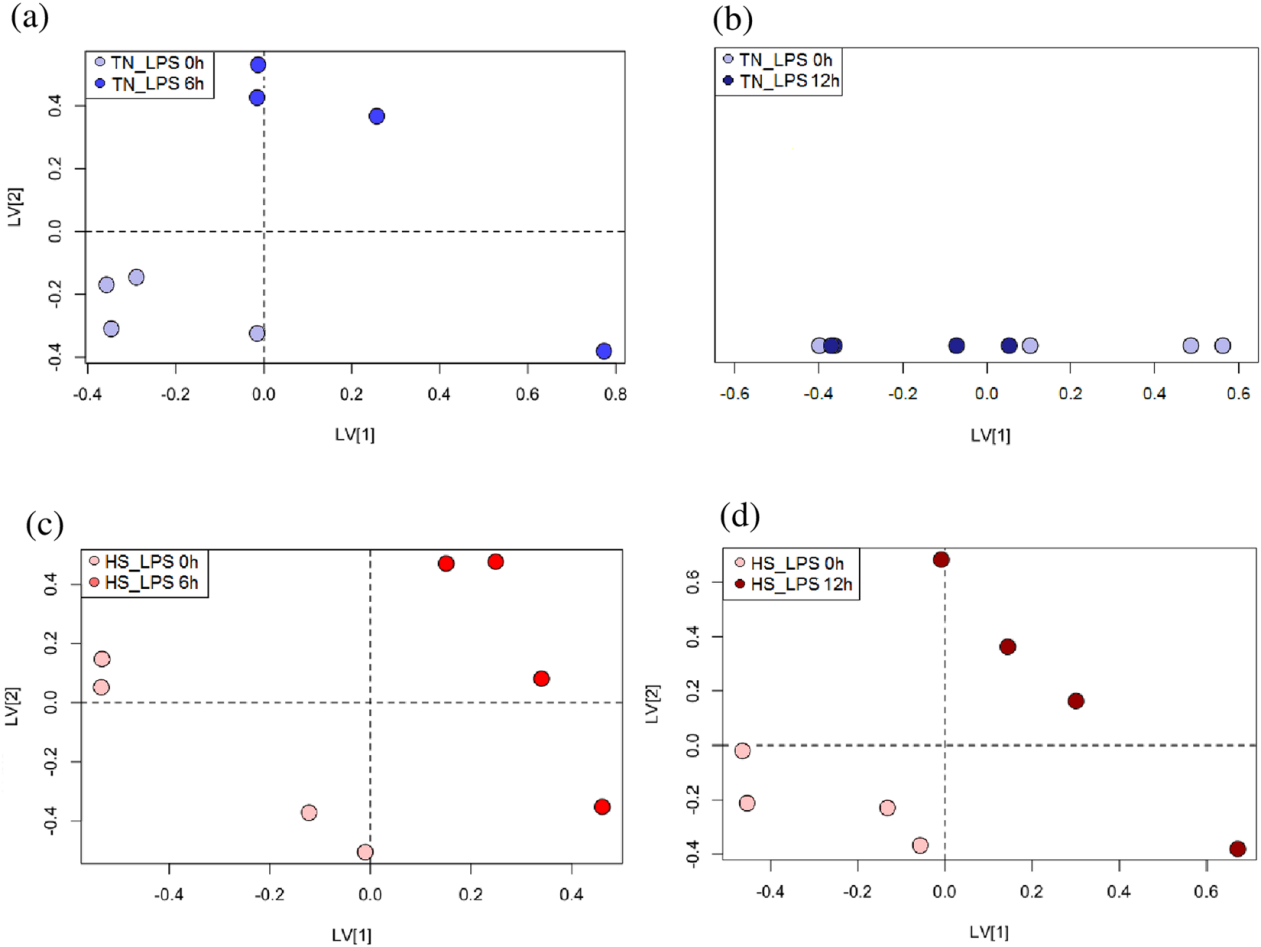
Partial Least Squares-Discriminant Analysis (PLS-DA) scores plot of ^1^H NMR-milk metabolomics spectra in dairy goats maintained under thermal neutral (TN; n = 4) or heat stress (HS; n = 4) for 15 days. On day 12, one half-udder in each TN and HS goat was administered with 10 μg *E. coli* lipopolysaccharide (LPS) or saline as the control (CON). The comparisons at different time points were as follows: (**a**) TN-LPS at 0 h vs. at 6 h, (**b**) TN-LPS at 0 h vs. at 12 h, (**c**) HS-LPS at 0 h vs. at 6 h, and (**d**) HS-LPS at 0 h vs. at 12 h.

Milk metabolomic profile of CON and LPS udder halves in TN (TN-CON vs. TN-LPS) and HS (HS-CON vs. HS-LPS) goats were analysed at 0, 4, 6, 12, and 24 h. The principal component analysis revealed that no outliers were found in the fitted model. The PLS-DA scores plot showed stronger separation between time points in HS-LPS compared to TN-LPS halves because of overlapping between initial and final time points in case of TN-LPS treatment. The comparisons 0 vs 6 h and 0 vs 12 h are shown in Fig. 4 for both TN-LPS and HS-LPS goats. The best cross-validated PLS-DA model for TN-LPS udders at 6 h post-challenge was obtained using the first 2 latent variables (*R*^2^_*x*_ = 0.74, *R*^2^_*y*_ = 0.11 and *Q*^2^ = 0.68; Fig. 4a), whereas TN-LPS udders at 12 h was not predictive and was given only for one latent variable (*R*^2^_*x*_ = 0.60 and *Q*^2^ = −0.50; Fig. 4b). On the other hand, a significant regression with 2 latent variables were observed in PLS-DA model for HS-LPS at 6 h (*R*^2^_*x*_ = 0.51, *R*^2^_*y*_ = 0.28 and *Q*^2^ = 0.75; Fig. 4c) and was still significant at 12 h post-challenge (*R*^2^_*x*_ = 0.80, *R*^2^_*y*_ = 0.07 and *Q*^2^ = 0.78; Fig. 4d). This difference between TN and HS in response to LPS over time can be explained by the fact that metabolites were generally less affected and restored earlier in TN than in HS conditions as discussed above.

**Figure 5:**
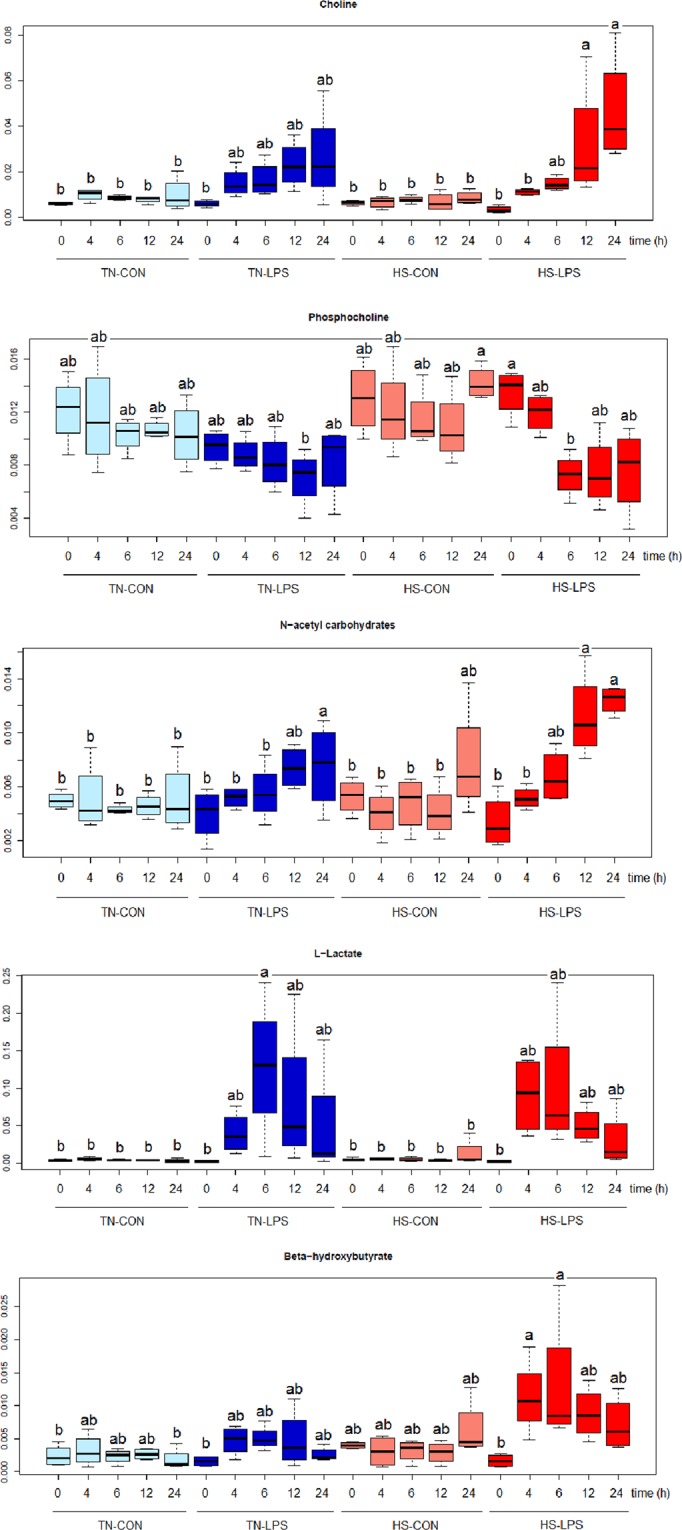
Hourly means and SEM for milk metabolites detected by ^1^H NMR-based metabolomics. The metabolomic profile was evaluated following intramammary injection of endotoxin in dairy goats maintained under thermal neutral (TN; n = 4) or heat stress (HS; n = 4) for 15 days. On day 12, one half-udder in each TN and HS goat was administered with 10 μg *E. coli* LPS (LPS), whereas the other half was injected with saline as the control (CON). This resulted in 4 treatment combinations: TN-CON, TN-LPS, HS-CON, and HS-LPS. ^a,b^means with different superscript differ (*P* < 0.05).

The top-ranking chemical shifts responsible for discriminating time points after LPS administration were lactose (*δ* 3.84), choline (*δ* 3.19), phosphocholine (*δ* 4.16), N-acetylcarbohydrates (*δ* 2.05), L-lactate (*δ* 1.32), and ß-hydroxybutyrate (*δ* 1.20), with different change profile over time between TN and HS goats (Fig. 5).

Lactose detected by ^1^H NMR metabolomics decreased after LPS infusion, which agrees with changes observed in this component by the chemical analysis of milk (Fig. 2c).

Generally, metabolites did not change (*P* > 0.15) over time in udder halves without LPS treatment (TN-CON and TN-LPS). Choline levels in milk increased in HS-LPS udders throughout time, reaching its maximum values at 12 (*P* < 0.05) and 24 h (*P* < 0.01) post-challenge (Fig. 5). On the contrary, phosphocholine (Fig. 5) decreased its levels in the HS-LPS udders at 6 h (*P* < 0.05) and tended to be different at 12 and 24 h (*P* < 0.10). Both choline and phosphocholine levels in milk of TN-LPS followed similar pattern over time as in HS-LPS, but the changes were not significant (*P* > 0.34).

Although choline can be synthesized in the liver and released into blood, a correlation between plasma free choline and milk choline was not observed in cows^42^, suggesting that at least part of the choline found in milk is synthesized in the mammary gland. Differences in milk choline after LPS treatment of HS goats in the current study might be related to a cholinergic signalling in the mammary gland immune system induced by LPS. In fact, choline has been described as a biomarker of inflammation in breastfeeding women, where a strong positive correlation was detected between choline and serum C-reactive protein levels^43^. However, mammary tight junctions become leaky in response to LPS treatment, and HS might have aggravated the degree of leakiness as mentioned above. Consequently, the possibility that some choline in milk was moved from blood to milk in HS goats cannot be precluded.

As shown in Fig. 5, an increase (*P* < 0.05) in milk N-acetylcarbohydrates levels were detected after LPS injection in both TN and HS conditions. N-acetylcarbohydrates are oligosaccharides synthesized and secreted by the mammary epithelial cell^44^. The N-acetyl carbohydrates have anti-adhesive effects on bacterial, viral and protozoa pathogens, preventing them from binding to mammary tissue. It seems that N-acetylcarbohydrates increased in the current study to boost the mammary immune system against the simulated infection by LPS.

L-lactate was increased by LPS injection significantly in the TN, but not in the HS udder halves (LPS × HS interaction; *P* < 0.05). L-lactate peaked at 6 h post-challenge (*P* < 0.05) and then returned progressively to its basal level in TN-LPS udder halves. The LPS induces formation of L-lactate by acute shift of epithelial cell metabolism from mainly mitochondrial-oxidative to principally glycolytic, which helps in meeting the increased energy requirements of the immune system when infection occurrs^45^. Increment in milk L-lactate after LPS challenge or natural infection has been reported in dairy cows^45^.

Milk ß-hydroxybutyrate (BHBA) increased at 4 (*P* < 0.05) and 6 h (*P* < 0.01) after LPS injection in HS, but not in TN conditions (LPS × HS interaction; *P* < 0.05; Fig. 5). The BHBA could be used as a source of energy to spare glucose under heat stress conditions^1^. For the immune cells, although glucose is the most quantitatively important fuel, there are other fuels such as glutamine, fatty acids, and ketone bodies^46^. In the current study, we observed that changes in energy sources by heat stress might have resulted in a shift in the fuel source of immune cells. Immune cells in TN goats mainly used glucose as energy source and metabolized it to L-lactate, whereas under HS conditions BHBA together with glucose were important energy sources (Fig. 5). Although heat-stressed dairy goats suffer negative energy balance, blood levels of non-esterified fatty acids do not change, but blood BHBA values increase consistently^1,4^. Thus, it is also possible that the increment in BHBA in milk of HS-LPS goats is due to the movement from blood thorough the leaky mammary tight junctions.

Correlations between the putative inflammation markers in milk detected by NMR and milk components during the first 24 hours of LPS treatment are shown in Table 3. With regard to correlation of milk metabolites with SCC, choline, acetylcarbohydrates and lactate were correlated positively (*P* < 0.05), whereas phosphocholine was correlated negatively (*P* < 0.05) in both TN and HS goats. On the other hand, milk BHBA was correlated positively (*P* < 0.01) with milk SCC in HS but not in TN goats. As discussed in the previous paragraph, BHBA could be used by immune cells as an energy source together with glucose in case of HS, whereas glucose is the main energy source under TN conditions. Sundekilde *et al*.^47^ applied H-NMR to compare milk metabolomics between milk with high and low SCC in dairy cows. They detected 8 metabolites (acetate, BHBA, butyrate, fumarate, hippurate, isoleucine, lactate, and lactose) that were greater in milk produced from udders with high SCC. Three of these metabolites (lactate, lactose and BHBA) were detected in the current study as putative inflammation markers in addition to other metabolites. We should keep in mind that in the study of Sundekilde *et al*.^47^ on dairy cows, milk with high SCC came from subclinical infections caused by a variety of different microorganisms, and thereby metabolite markers were originated from those heterogeneous pathogens, immune cells and mammary tissue. Our study, on the other hand, was carried out on goats and metabolites were exclusively originated from immune and mammary cells. In contrary to our results and the results of Sundekilde *et al*.^47^, Klein *et al*.^48^ found no correlation between milk SCC and lactate in dairy cows.

**Table 3:** Correlations between milk putative inflammation markers identified by NMR and milk components in dairy goats exposed to thermal neutral (TN; n = 4) and heat stress (HS; n = 4) conditions for 15 days.

Metabolite	Fat		Protein		Lactose		SCC	
	TN	HS	TN	HS	TN	HS	TN	HS
Choline	0.163	−0.016	0.698^***^	0.180	−0.318^*^	−0.055	0.560^***^	0.370^*^
Phosphocholine	−0.152	−0.110	−0.639^***^	−0.179	0.281	0.378^*^	−0.524^***^	−0.396^*^
Acetylcarbohydrates	0.112	0.364^*^	0.407^**^	0.425^**^	−0.180	−0.334^*^	0.461^**^	0.510^***^
L-lactate	−0.007	−0.021	0.543^***^	0.610^***^	−0.783^***^	−0.743^***^	0.500^***^	0.501^***^
ß-hydroxybutyrate	0.165	−0.009	0.396*	0.608^***^	−0.642^***^	−0.761^***^	0.288	0.498^**^

For each TN and HS goat, one half-udder was administered with 10 μg *E. coli* lipopolysaccharide (LPS), whereas the other half was injected with saline as the control (CON) at day 12 of the thermal treatment.

*P < 0.05, **P < 0.01, ***P < 0.001.

To our knowledge associations between milk metabolome and SCC or other milk components have not been evaluated in dairy goats under TN and HS conditions. As shown in Table 3, the magnitude of correlations varied according to ambient temperature. Thus, some metabolites correlated with some milk components in TN but not in HS and vice versa. For instance, significant correlations between choline and milk protein as well as lactose were detected in TN only. Nevertheless, both phosphocholine and acetylcarbohydrates correlated with milk lactose only in HS goats. This clearly indicates differences in inflammation kinetics according to the ambient temperature.

## Conclusions

Heat stress caused marked changes in thermophysiological traits in dairy goats, including greater rectal temperature and respiratory rate, alongside decreased feed intake and milk yield. The fact that the systemic febrile resulting from LPS administration was masked by the greater body temperatures in HS goats makes it difficult to detect infections under such conditions. Somatic cell recruitment after intramammary endotoxin challenge was delayed in heat-stressed goats. Heat treatment resulted in greater milk citrate concentrations. Milk choline, phosphocholine, N-acetylcarbohydrates, L-lactate and ß-hydroxybutyrate were identified as putative mammary inflammation markers. However, the importance of these putative markers varied between TN and HS indicating different mammary immune response. Overall, heat stress negatively affected the mammary immune response, which could make HS animals more prone to mastitis.
